# Using a Combined Lean and Person-Centred Approach to Support the Resumption of Routine Hospital Activity following the First Wave of COVID-19

**DOI:** 10.3390/ijerph19052754

**Published:** 2022-02-27

**Authors:** Ailish Daly, Sean Paul Teeling, Suzanne Garvey, Marie Ward, Martin McNamara

**Affiliations:** 1 Beacon Hospital, Beacon Court, Bracken Road, Sandyford Business Park, Sandyford, Dublin 18, D18 AK68 Dublin, Ireland; suzanne.garvey@beaconhospital.ie; 2 UCD Centre for Interdisciplinary Research, Education & Innovation in Health Systems, Midwifery & Health Systems, UCD Health Sciences Centre, School of Nursing, D04 V1W8 Dublin, Ireland; sean.p.teeling@ucd.ie (S.P.T.); martin.mcnamara@ucd.ie (M.M.); 3 Centre for Person-Centred Practice Research, Division of Nursing, School of Health Sciences, Queen Margaret University, Musselburgh EH21 6UU, UK; 4 Centre for Innovative Human Systems, School of Psychology, Trinity College, The University of Dublin, D02 PN40 Dublin, Ireland; marie.ward@tcd.ie

**Keywords:** COVID-19, acute hospital, Lean, rapid improvement event, person-centred

## Abstract

The unexpected advent of the COVID-19 pandemic led to a sudden disruption of routine medical care, with a subsequent reorganization of hospital structures and of care. Case studies are becoming available in the literature referring to the logistical difficulties involved in a hospital resuming normal activity following the first COVID-19 lockdown period. This paper details the experience of a study site, a private hospital in Dublin, Ireland, in the redesign of service delivery in compliance with new COVID-19 prevention regulations to facilitate the resumption of routine hospital activity following the first wave of COVID-19. The aim was to resume routine activity and optimize patient activity, whilst remaining compliant with COVID-19 guidelines. We employed a pre-/post-intervention design using Lean methodology and utilised a rapid improvement event (RIE) approach underpinned by person-centred principles. This was a system-wide improvement including all hospital staff, facilitated by a specific project team including the chief operation officer, allied therapy manager (encompassing health and social care professionals), infection prevention and control team, head of surgical services, clinical nurse managers, patient services manager and the head of procurement. Following our intervention, hospital services resumed successfully, with the initial service resumption meeting the organizational target of a 75% bed occupancy rate, while the number of resumed surgeries exceeded the target by 13%. Our outpatient visits recovered to exceed the attendance numbers pre-COVID-19 in 2019 by 10%. In addition, patient satisfaction improved from 93% to 95%, and importantly, we had no in-hospital patient COVID-19 transmission in the study period of July to December 2020.

## 1. Introduction

The COVID-19 pandemic presented health services across the globe with an unprecedented need for rapid changes to how services were delivered, and created a number of ethical dilemmas, particularly for those providing direct clinical care [1,2,3]. Never before had health services had to change so quickly, with healthcare organizations around the world facing unprecedented challenges in responding to the first wave of the COVID-19 pandemic. The unexpected advent of the pandemic led to a sudden disruption of routine medical care, with a subsequent reorganization of hospital structures and foci of care. The management of increased volumes of acutely unwell patients challenged even the most advanced healthcare providers. Lessons learnt around the clinical management/acute response to a pandemic will continue to be gathered as well as data around patient recovery and the long term clinical impact of the pandemic [1,2,3]. During the initial COVID-19 wave, many hospitals, in response to new health demands on capacity, postponed elective (scheduled) procedures and what were deemed as non-essential services [4]. Internationally, the move to return to a pre-COVID-19 level of routine elective services after they were suspended or postponed has proven to be a challenge for healthcare institutions [5]. For example, the repurposing of clinical spaces in response to the COVID-19 left hospitals with less capacity for routine activity. Case studies are becoming available in the literature, referring to the logistical difficulties involved in a hospital resuming normal activity following the first COVID-19 lockdown period [6,7,8]. Additionally, research is now being undertaken into the impact of COVID-19 not just on staff physical health, but on its considerable impact on the psychological wellbeing of healthcare staff [9]. During the first wave of the pandemic, the impact on the physical health of healthcare staff was noted with healthcare workers disproportionately affected by COVID-19, as 32% of cases were detected in healthcare staff internationally. Within the health system in Ireland, pressures were noted in the percentage of patients requiring admission to hospital (12.9%) and also in intensive care (1.9%) [10]. By the end of December 2020, there were nearly 92,000 positive cases of COVID-19 recorded in Ireland, with almost 700 of these cases admitted to an intensive care unit (ICU). More than 2200 people had died from COVID-19 [11]. Prior to the COVID-19 pandemic, the National Adult Critical Care Bed Capacity was 255 beds [12].

Many healthcare staff are trained and experienced in quality and process improvement methodologies which have been used to respond to the new challenges of working in the COVID-19 era [13]. This paper describes the utilisation of Lean process improvement methodology to support the resumption of activity of a private hospital in Ireland as part of a recovery plan following the first wave of COVID-19 in July 2020, taking into account the multiple challenges encountered. Challenges faced were similar to those faced by every healthcare organisation and are still apparent—how to deal with an increased volume and management of patient presentations, complicated by a delay in accessing healthcare due to initial lockdown, and how to support staff in continuing to deliver person-centred care in a vastly changed environment [8,14]. The organisation recognised the complexity of these challenges and chose to utilise a combined Lean and person-centred approach in planning the resumption of services following the first COVID-19 lockdown. Both Lean and person-centred methodologies have been shown to be synergistic approaches for process improvement [15,16,17]. The organisation had, in 2016, committed to a whole-system approach to process improvement with a focus on using person-centred, Lean, Six Sigma and Lean Six Sigma (LSS) methodologies [18].

Through understanding culture, system function, action triggers and sensemaking, the organisation had embarked on a process to devolve power and influence from the executive level and create a network of improvement specialists at all levels in the organisation [18].

### 1.1. Background

The study site is an Irish private hospital. A private hospital indicates the organisation operates independently of the state health services and receives no state funding. Care is funded through private health insurance. Public health services in Ireland are provided in health service executive (HSE) hospitals as well as public voluntary hospitals. The hospital provides services across all specialties, including oncology, orthopaedics, general medicine, general surgery, intensive care, emergency medicine, and paediatrics as well as all supporting services. The hospital employs 1500 healthcare staff with 800 consultants. As with every healthcare organisation, the delivery of care changed dramatically in March 2020.

Private hospital status indicates that the organisation operates independently of state health services and receives no state funding. Care is funded through private health insurance. Public health services in Ireland are provided in health service executive (HSE) hospitals and public voluntary hospitals and, in practice, there is very little difference between these two types of hospitals [19]. Of note, many of these hospitals also provide private health care, but they must clearly distinguish between public and private beds [19].

Ireland entered a national lockdown and citizens, unless classified as essential workers, were asked to stay at home [20]. Only essential healthcare appointments were permitted from March 2020 to June 2020. The objective of this was twofold. Firstly, minimizing population circulation would lead to a reduced risk of transmission of COVID-19. Secondly, by minimizing non-essential healthcare, capacity was created to care for patients who had contracted COVID-19. As COVID-19 was an unknown variable that was rapidly spreading, the capacity of the acute hospital system to care for the numbers of patients who would require hospital admission to general or intensive care services was a concern [10]. Therefore, the Irish government and public health service executive agreed with private hospitals to “takeover” the capacity in the private hospitals. Private hospitals effectively became a part of the public health system from 19 March to 30 June 2020. The expectation was that this acute hospital capacity would be used for intensive care/high dependency management of COVID-19 patients, as well as to facilitate urgent time-dependent surgery for non-COVID-19 patients. As Ireland’s COVID-19 rates stabilized in June 2020, it was clear that this capacity was no longer required. The agreement between the public health system and private hospitals was stood down. The private hospitals started to plan to return to “normal activities” in a very new environment. The purpose of this paper is to examine the processes used to plan and execute a rapid but smooth transition to normal activity following the first wave of the COVID-19 pandemic within the study site.

The study site, in conjunction with its academic partner, University College Dublin (UCD), hosts an education and training academy that, as a part of its continuing goal to deliver excellent patient care, in 2017, initiated an education and training programme in Lean and Six Sigma to support staff in delivering high-quality care. Lean is an approach to improving organizations that focus on the needs of customers, and considers everything that is neither delivering value to customers nor ensuring the safety and security of the organisation and its staff as ‘waste’ and therefore a target for elimination [21,22]. Six Sigma aims to reduce variation in a process [21]. A combination of both methodologies, Lean Six Sigma (LSS) has been used in healthcare since the early 2000s with the aim of improving efficiency and achieving quality and operational excellence [23,24]. As healthcare providers worldwide, whether publicly or privately funded, are faced with similar challenges of caring for an ageing population with a limited pool of financial and personnel resources, the need to seek efficiencies while continuing to provide quality services has become more and more acute [25]. LSS has been implemented in many healthcare organizations with improvements achieved across many clinical and administrative pathways and processes, including medication management [26,27], specific patient conditions, such as stroke and dementia [28,29], and theatre organisation, efficiency, and patient flow projects [30]. McCormack and McCance (page 3) [31] define Person-centred care as “an approach to practice established through the formation and fostering of healthful relationships between all care providers, service users and others significant to them in their lives. It is underpinned by values of respect for persons (personhood), individual right to self-determination, mutual respect, and understanding. It is enabled by cultures of empowerment that foster continuous approaches to practice development”. There is a growing body of work indicating the synergistic use of LSS and person-centred approaches to improve processes in healthcare settings [15,16,17].

By 2020, the education and training programme at the study site had matured to a team of 13 advanced qualified process improvement practitioners who had completed a post-graduate certificate or diploma training in process improvement in health systems. These practitioners had previously led and delivered process and quality improvement projects across a wide variety of hospital services, including the delivery of mandatory training to healthcare staff [32], streamlining of booking of elective surgeries [33], procurement and operating room stock management [34] and releasing operating room nursing time to care [35]. The process improvement practitioners within the hospital, with their experience of leading and delivering change, were therefore highly involved in the work of the study site to resume routine activity, with process improvement practitioners internal to an organisation shown to be well received by and supportive of staff [15,17].

### 1.2. Objective

The objective of this improvement project was to resume routine service after the first COVID-19 lockdown in Ireland, the time period of which was from March to June 2020. A successful resumption of services would require putting in place correct and safe procedures to allow our patients to attend their appointments with a reliance on external factors ranging from government and department of health guidelines to issues of supply chain management. The organisation would also have to ensure staff across all hospital departments from patient-facing to administration were supported and safe in their return to delivering and supporting care. Key requirements identified by hospital management and infection prevention and control teams in line with government guidance were ensuring that the following were available:

1. COVID-19 swabbing and a testing facility for patients due to attend for general anaesthetic;
2. Correct personal protective equipment (PPE) and training in its use for all staff at the point of use (POU);
3. Education in and the implementation of prescribed 2 m social distancing rules;
4. COVID-19 screening questionnaires and temperature checks to identify symptomatic patients and staff members and implement isolation and testing;
5. A process for assessment of symptomatic or close contact team members.

Success would be measured by the following metrics:

- Patient attendance (figures to return to 75% of normal activity by 1 September 2020, date). The target of 75% was agreed as feasible by the executive management team. This reflected decreased activity during the period that normal hospital services were paused from March to June 2020;
- Patient feedback on their hospital experience;
- Hospital performance—the number of outpatient appointments provided, number of surgeries, and inpatient occupancy versus the 2019 performance. These metrics were the standard key performance indicators of activity within the study site;
- Quality indicators: COVID-19 transmission rates among patients. Adherence to the infection prevention and control (IPC) parameters;
- Staff engagement and feedback.

## 2. Methodology and Methods

In June 2020, as the organisation commenced planning a return to routine activity, the challenges that lay ahead were clear for all to see, however, the solutions were not. We used a pre-post study design to inform our work. A pre-post study design measures a variable of interest before and after an intervention in the same location, setting and participants [36]. For this study, a pre-/post-intervention design was employed using Lean methodology to measure variables related to the resumption of normal hospital activity following COVID-19 lockdown. The design enabled us to measure the impact of a Lean redesign of existing processes for the resumption of normal services across all hospital departments. The study site had a successful record of incremental and sustainable improvement using both Lean and Six Sigma methodologies supported by person-centred approaches. Improvement work routinely used the LSS define, measure, analyse, improve, control (DMAIC) framework to structure the improvement through process redesign [18,32,33,34,35,37]. The organisation had also used the LSS define, measure, analyse, design, validate (DMADV) framework [38] to co-design new processes. However, on this occasion, due to the urgency and pressure created by the evolving COVID-19 pandemic, it was decided to use a more rapid approach. Six Sigma’s data-driven approach, whilst providing the statistical evidence for change, has a potential for what has been called “analysis paralysis” [39,40], where a large amount of time and human resources are spent collecting and analyzing data. The organisation therefore decided to focus on a more rapid process improvement facilitated by Lean methodology and using a Lean rapid improvement event (RIE) framework to structure the improvement [22]. The key improvement tools used throughout the improvement process are set out in Table 1.

Tools were chosen for both functionality and user-friendliness as they were to be deployed across all disciplines and departments, with staff supported by qualified process improvement practitioners who themselves were members of staff based within the study site. The rapid approach enabled us to involve cross-functional teams, solution generation and implementation in seven days (Table 2).

### 2.1. Methods

Following the first wave of COVID-19, when the hospital management within the study site was first notified that the agreement with the public health system was to be stood down, the CEO and executive management team met with the wider management group to discuss resuming hospital services. The challenges ahead, potential barriers and concerns were discussed, and future state targets were identified as:

1. Resumption of normal service delivery measured by:
  - Inpatient occupancy comparable to the 2019 rate of 88%;
  - Number of surgeries comparable to the 2019 figure of 17,378;
  - Number of outpatient visits compared to the 2019 figure of 112,906.
2. Safe completion of service resumption through minimizing COVID-19 transmissions, measured by infection rates among patients per month for the period from June 2020 to December 2020.

Recognising the complexity of this task, it was agreed by the executive management team to convene a resumption of service project team, the membership of which is outlined in Table 3.

#### 2.1.1. Ways of Working

In considering the membership and operations of the team, key considerations were local knowledge of hospital operations, expertise in process improvement and project management, knowledge of government guidance and policy in infection prevention and control, as well as specific procurement expertise. Recognising the challenge of the urgency of the project, combined with a motivation for resumption to normal activity, and being acutely aware of staff worries and concerns, each project team member committed to using person-centred ways of working throughout the project. Person-centred approaches to improvement have a strong focus on the concept of respect for persons, which underpins both person-centredness and Lean approaches to improvement [15,17]. Additionally, person-centredness emphasizes the development of person-centred cultures through the use of collaborative, inclusive, and participatory (CIP) principles [41]. The executive management team moved from a “power” culture of command and control to a “task” culture where team members and stakeholders were enabled to explore challenges, voice concerns and co-create solutions [42]. Adapting a collective and co-design approach has been shown to improve team performance and safety in hospitals [43]. The project team agreed to use co-design principles, and invited the stakeholders to work in collaborative, inclusive and participative teams. Having established targets and operating principles, the project team agreed to utilise a Lean rapid improvement event approach for this process improvement.

#### 2.1.2. Rapid Improvement Event (RIE)

Eaton, page 145 [22], explains, “A rapid improvement event focuses the effort of a group of people for a finite period of time on a defined problem, at the end of which something has changed”.

The RIE is an approach developed in the industry by Toyota that can be utilised to establish sustained improvements in complex environments, with healthcare recognised as being the most complex of any service industry environment [44]. The process is also known as a kaizen or kaizen blitz, as it is designed to rapidly assess a defined problem and co-design changes to address it [45]. Kaizen, in English, means “good change” and originates in the three main features of Japanese management philosophy, which are harmony and loyalty, consensus in decision-making and employment for life [15,17,46]. Kaizen is a standard approach to team-based problem-solving in Lean, with the improvement conducted by improvement teams to implement improvements quickly in a specific area [47]. Importantly, congruent with our goal to ensure staff were supported and safe in their return to routine activity, kaizen has been shown to be synergistic with person-centred approaches to change in health systems [15,17]. The kaizen event typically is completed in seven days or less, depending on the local context. In the study site, RIE took place over a seven-day period (Table 2).

Following this, the project team identified the key customers/stakeholders as predominantly patients, hospital staff and consultant teams, and general practitioners (GPs).

The project team agreed that a person-centred approach would be taken when planning the resumption of normal service. Adopting a person-centred approach requires respecting the needs, preferences and concerns of the individual, listening to their voice, learning what is the value add/non-value add and ensuring individuals are empowered to voice concerns and seek solutions [48]. In empowering the individual to voice concerns and seek solutions, a collective leadership approach was taken to implementing change. Changes were based on the collective leadership pillars of performance, safety, wellbeing, team process and sustaining improvement [43].

#### 2.1.3. Planning for the RIE

The service resumption team recognised from the outside that this project would be unique in its complexity and would require input from many disciplines across the hospital. The RIE made use of identified key improvement tools (Table 1) to support stakeholder engagement, and the process improvement practitioners within the hospital were available to support staff in their use.

The group used a SIPOC [21] chart to identify all the stakeholders whose expertise and support would be required. The SIPOC (Table 4) enabled:

1. A high-level view of the process for patients and staff in attending the hospital.
2. Identification of stakeholders in their role as providing or attending hospital appointments.
3. The inputs the stakeholders had into the process.
4. The expected outputs from the process.

**Figure 1:**
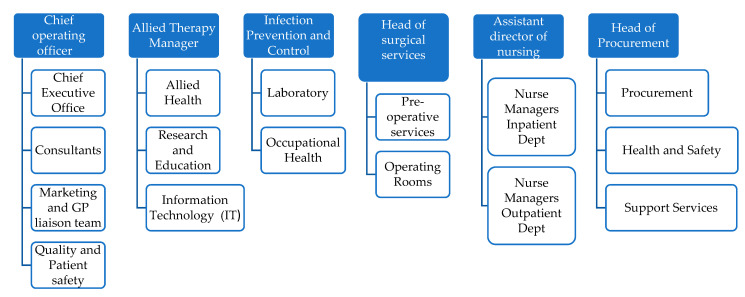
Stakeholder Tree.

The theme of stakeholder engagement has been widely discussed in the context of health systems and healthcare organizations [49,50,51,52]. The importance of an understanding of the positionality of stakeholders in hospitals has also been recognised as making a significant contribution to organizational change [53]. Due to the volume of stakeholders involved in the resumption of routine activity, one of the priorities of the project team was to ensure that each stakeholder had a “seat” at the project table, therefore each member of the project team (Table 3) was designated as a link person to a group of stakeholders (Figure 1). The project team also utilise a responsible, accountable, inform, communicate (RACI) matrix that clarifies responsibilities, tasks or deliverables to cross-functional teams and projects that involve many departments [54]. Together, the RACI and SIPOC facilitated a comprehensive stakeholder engagement.

#### 2.1.4. Voice of the Customer

The initial engagement was carried out by a small group voice of the customer/breakout sessions (*n* = 10) that were facilitated by the improvement practitioners and service resumption project team members. Hospital management asked that all hospital departments attend and participate. This reflected the importance of collectively identifying challenges and co-designing solutions. The terminology voice of the customer (VoC) is used to denote the expectations of the customer [48]. Valuing the person as an expert in their life experience and respecting this by considering the whole person is kept to the fore in process improvement by listening to the “voice of the customer” [15,17,55]. To capture the voice of staff, within our workshops, we commenced brainstorming sessions with affinity diagrams [56] with the intention of generating, organizing and categorizing a large volume of ideas around focused topics. The use of the affinity diagrams indicated areas for focus related to COVID-19 swabbing of patients due for admission to the hospital, COVID-19 screening of patients due to attend for an outpatient appointment as well as patients due for hospital admission. Related factors included PPE, Social Distancing, Staff transmission, Staff welfare, Patient Safety.

Our voice of the customer findings from our brainstorming revealed the main concerns of staff. These concerns are presented here in order of the highest frequency of mention and discussion at our workshops.

- Risk of transmission between staff and from staff to patient/patient to staff.
  Concerns expressed included:
  “I live with elderly parents–I don’t want to bring infection risk home”;
  “I have an underlying condition and am anxious about working in small office space”.
- Management of patient volumes:
  “Managing our usual volumes and maintaining social distance will be a challenge”.
- Process for referral and reporting of COVID-19 Swabs:
  “Who needs to be swabbed”;
  “who tells the patient or staff member their result”;
  “Consultant *** requests that all his patients are swabbed …”
- Process for COVID-19 Screening:
  “What happens if I don’t complete the COVID-19 screen–can I still attend?”
- Absence management:
  “What happens if 2 of my team members are asked to isolate”.

This customer voice, elicited through our brainstorming, revealed commonality among stakeholders regarding the resumption of service themes and concerns, as well as opportunities for standardised answers or processes to address these concerns. We followed these voice of the customer sessions with open flow brainstorming via workshops. These workshops used our agreed collaborative, inclusive and participatory (CIP) principles that have been shown to facilitate participant feedback and enable a thematic analysis of findings. Stakeholders contributed their ideas as to what changes or improvements could be implemented to complete the hospital resume services. Thematic analysis is the process of identifying patterns or themes within qualitative data and, according to Braun and Clarke [57], is sufficiently flexible to support the analysis of data collected from interviews, focus groups, workshops, meetings or surveys. The results of the workshops were thematically analyzed by participants into four themes:

1. Patient safety and care;
2. Staff support;
3. PPE;
4. Social distancing.

**Figure 2:**
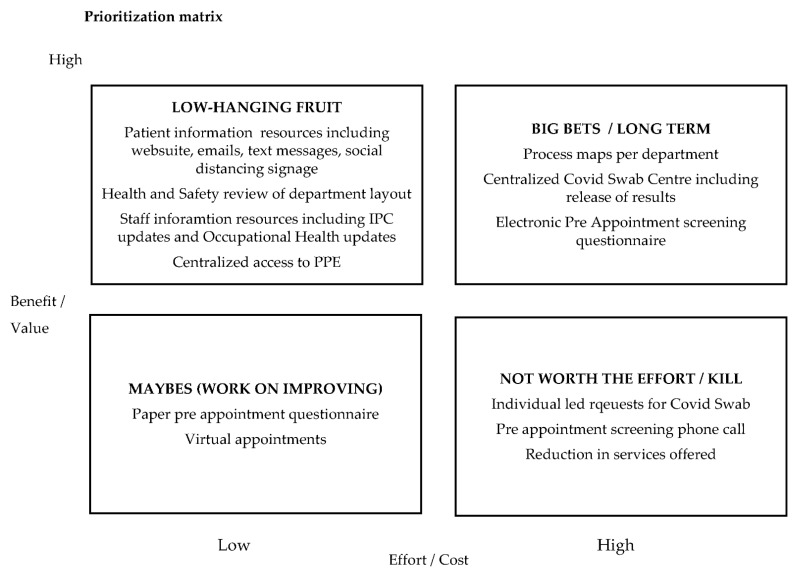
PICK chart of suggested improvements.

From the identified themes, a PICK (possible, implement, challenge, kill) [35,58] chart (Figure 2) was used to classify the improvements required, essentially to identify low hanging fruit versus more complex changes [50]. Using a high/low cost/benefit scoring matrix inherent in the PICK chart solutions were categories into quick wins (low effort with high payoff), short term solutions that would require more work (low effort, low reward) and long term solutions that would require a high effort but would bring high reward. Solutions that would require high effort and bring little reward were also classified, and an agreement was reached not to pursue.

The collaborative PICK chart process allowed stakeholders to consider solutions from each other’s perspectives. For example, the suggestion of a pre-appointment screening phone call for each patient was considered a solution to assessing patient COVID-19 transmission risk factors. However, the presence of patient services in the improvement workshops allowed their voice to be heard and for them to advise the wider team that the time taken to complete phone calls for one consultant clinic alone was on average 3 h of patient services time. Scaling this up for a department that runs 10 consultants’ clinics per day was impossible. This solution was classified as an excessively high effort and another solution was sought. The IT department was also included in stakeholder sessions and was then able to advise on the feasibility and time frame for implementing an electronic pre-appointment questionnaire to obviate multiple phone calls. Using a collaborative, inclusive approach where stakeholders co-created solutions allowed for an electronic pre-appointment COVID-19 screening questionnaire to be developed and implemented, ensuring patients attending for hospital appointments were screened for COVID-19 risk factors.

There is some discussion around the “not worth the effort”/“kill” section of a PICK chart [58]. Rather than discourage someone from offering an idea, there may be value in exploring the route of the idea and seeking the value/effort from their perspective. For example, rather than “killing” the idea of restricting non-essential services (therefore, reducing patient volumes through the organisation), as we explored the root of that idea, the issue was clarified, as the ability of the study site, to create extra space to accommodate high volumes of patients who had to social distance while attending high acuity critical services. The idea of restricting non-essential services was “killed”. However, other solutions were explored. An agreement was reached to extend the hours of operation across services. Accommodating the same number of patients over a longer period allowed for patients to attend and maintain social distancing in departments and allocate sufficient time for housekeeping between appointments. One potential concern with this solution was the impact of change of working hours on staff delivering care. Staff were empowered to participate in setting the hours of operations and rosters rather than a “top-down” approach to fostering a “bottom-up” approach where staff participated in creating rosters. Innovative solutions were found, including changes in shift pattern and flexibility in start/finish times, which have been adapted into long term service delivery in the hospital. 

#### 2.1.5. What We Implemented

Following our brainstorming and solution development, we implemented the following:

(a) High Reward/Low Effort Solutions

The collaborative and inclusive approach adapted from the outset of this RIE allowed the project team to implement the identified high reward/low effort (Figure 2) improvements within the seven-day RIE. With marketing and IT teams included as stakeholders from the outset, the content and delivery of electronic and paper information materials were agreed and in place without delay. Similarly, health and safety and procurement were on hand to follow through with immediate interventions in ensuring all departments had access to appropriate PPE as well as access to the health and safety review of the department layout, ensuring workspaces and patient treatment areas complied with social distancing regulations. By combining person-centred and Lean approaches, immediate interventions were possible [15], because we had consulted, listened to, and collaborated with our hospital team using CIP principles to support our use of Lean methodology.

(b) Short term low effort/low return solutions

Short term low effort/low return solutions, including paper-based pre-appointment screening and virtual appointments, were implemented to allow patients to attend appointments, however challenges around these solutions became apparent. Paper-based pre-appointment screening questionnaires contradicted the hospitals’ target to move to electronic patient records [33]. Moreso, the requirement to check these immediately before an appointment could result in appointment delays. Virtual appointments gave high returns in the initial lockdown period as patients could access healthcare personnel from home, however as the lockdown ended and patients had the option of attending appointments in person, most patients were keen to avail of this service, thus, the initial high return on relatively low effort reduced.

(c) Longer-term solutions

Having implemented “low hanging fruit and short term low effort/low return solutions” the group then focused on long term solutions for standardizing the process for patients and staff to safely attend the hospital. Long term solution planning was supported by comprehensive cross-functional process mapping for each department, detailing specific departmental processes for transitioning to the resumption of normal service. This reflected a complex task that would reach a range of departments, including those providing complex care for patients, such as the operating room (OR) or oncology day unit, as well as departments managing purely administrative tasks, such as patient accounts or research teams. A future state process map is a document for capturing and illustrating the anticipated better way to work [59]. It is used to move a process closer to the ideal state. To clearly define the future state, process maps were co-designed with local department managers to outline the specifics required for resuming service in their areas. These again were referred and influenced by the four identified focused themes from our workshops: patient safety and care, staff support, PPE and social distancing, that were reflected in these process maps.

**Figure 3:**
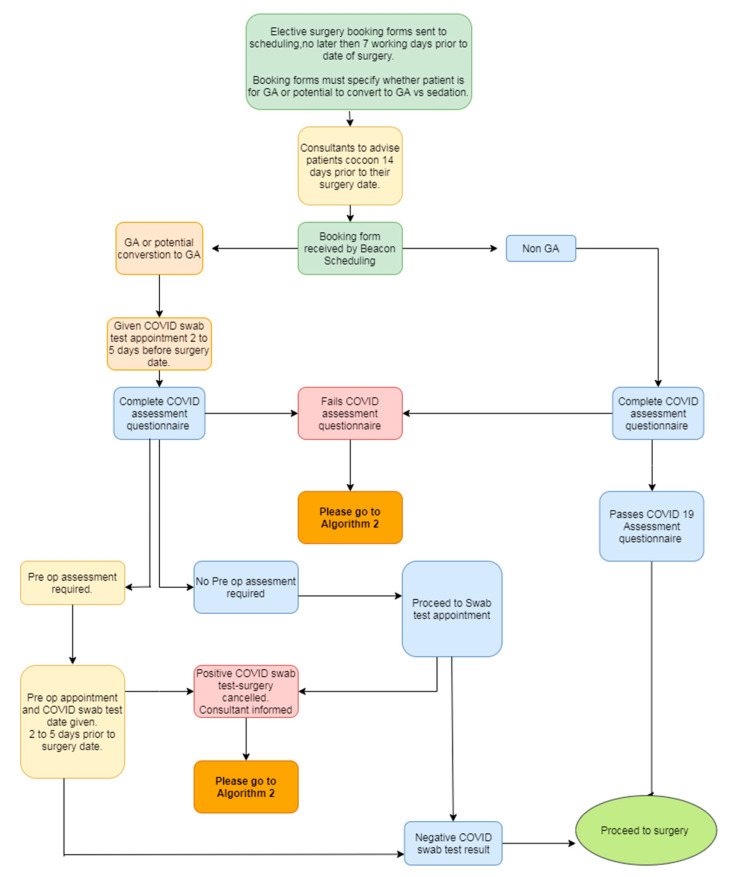
Process map of the journey of a patient attending for elective surgery.

**Figure 4:**
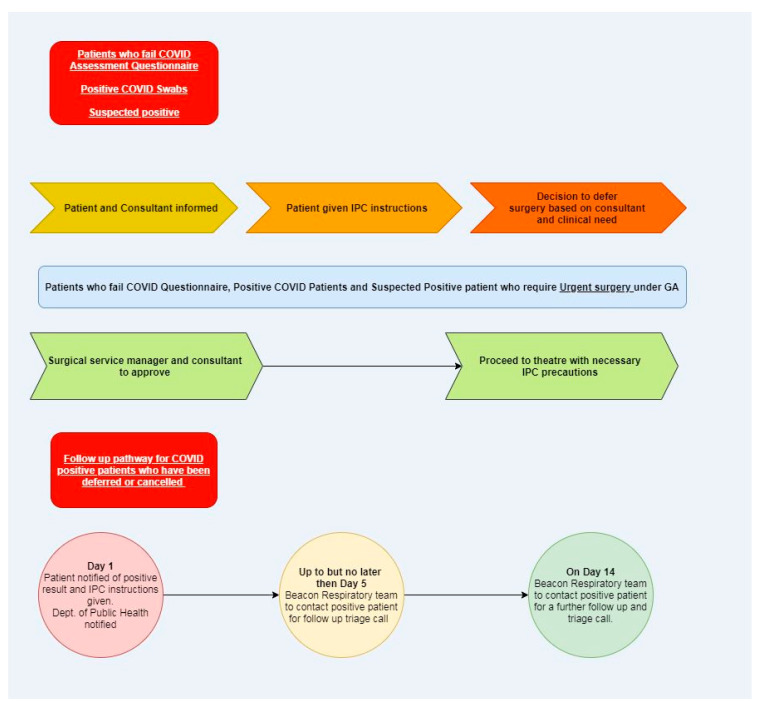
Algorithm for the management of failed COVID-19 screening questionnaire.

Process mapping workshops (*n* = 10) were completed for each department, by interdisciplinary teams within each department, and were facilitated by process improvement practitioners. Each team was asked to map the patient and staff journey through the department as services resumed, keeping in mind the key themes of COVID-19 swab requirements, COVID-19 screen requirements, PPE, social distancing and the management of symptomatic staff. PPE management, social distancing requirements and supports were addressed as “quick wins”. More complex, was achieving timely COVID-19 swabbing for patients before admission as well as completing a pre-appointment screening questionnaire for patients before attending a hospital appointment. Process mapping workshops quickly revealed the potential variance in how each department planned on managing these challenges. The working group quickly recommended and prioritized an implementation of an organisation-wide process for request, completion and reporting of pre-admission/pre-operative COVID-19 swabs through a dedicated team. An organisation-wide electronic pre-appointment screening questionnaire was implemented, including the process for the follow up of patients whose screen revealed a potential risk factor (recent history of international travel, new onset of cough or temperature, recent positive COVID-19 swab). Over 50 process maps were completed, reviewed, and validated by the project team. We detail a co-designed example of a process map for a patient attending elective surgery in Figure 3, and a developed algorithm for a failed COVID-19 assessment questionnaire or a positive COVID-19 swab, in Figure 4.

Key outputs of this process mapping exercise were:

1. An agreement on a standard process for all patients scheduled for or at high risk of conversion to general anaesthetic. This aspect of process mapping highlighted the potential for deviation across different departments where general anaesthesia (GA) was administered in a main operating room (OR), endoscopy department, or cardiac catheterization laboratories.
2. An agreed process for the management of a failed COVID-19 screen and failed COVID-19 swab. A person-centred approach was key to concluding this process. From the patients’ perspective, the acuity of the presenting complaint needed to be considered. In an emergent situation, a surgery may need to proceed despite a positive COVID-19 swab. Respecting the needs of the patient in facilitating the surgery and also the needs of the staff in ensuring correct and adequate PPE was in place and allowed an agreement on a process to facilitate urgent surgeries.
3. A team approach to process mapping sessions: Process mapping sessions were attended by members of the service resumption project team, process improvement practitioners and stakeholders from multiple hospital departments. This had two unexpected impacts.
  a Some staff had additional time to offer to support this process change, as activity in their relevant department had reduced due to COVID-19 lockdown.
  b However, activity in some high acute departments, such as ICU, general surgery and oncology increased during the lockdown period. The forum of the process mapping sessions allowed for a “levelling of the load”—stakeholders from across the hospital were able to learn about the potential challenges faced across departments and potential solutions—this reduced the potential of people working in silos and allowed for the cross-pollination of ideas.

Following the incremental completion of the voice of the customer, improvement prioritization, implementation planning and process mapping sessions, we had the following results, which we discuss below.

## 3. Results

Through our use of voice of customer to understand potential challenges, we had achieved visualization of process “pain” points/areas of non-value add. The opportunity to hear these challenges from the staff in the process across disciplines enabled the project team and staff to gain a system-wide understanding of potential difficulties ahead and take a collective approach to seek solutions [43]. Despite three months of reduced activity during COVID-19 lockdown, the organisation met or exceeded 2020 targets for all performance indicators agreed upon as the hospital transitioned to normal service in July 2020 (Table 5). Our occupancy rate of 75% was in line with the target while our number of surgeries exceeded the target. Our outpatient visits recovered to exceed the number provided pre COVID-19 in 2019. In comparison, outpatient attendances in acute public hospitals in 2020 were 2,992,016, which is below the 3,318,604 target. Surgical discharges from public hospitals dropped by 27% in 2020 versus 12% in the study site [60]. Comparing the period from July to December 2020 to the same period in 2019, inpatient admissions in the organisation increased by 6%, inpatient surgeries increased by 21% and outpatient surgeries increased by 4%. Nationally, in the period from July to December, surgical discharges dropped by 6% in the public hospital system [60]. In addition, patient satisfaction improved from 93% to 95%. Additionally, there was no in-hospital transmission from March 2020 through to December 2020. The risk of in-hospital transmission in the period from March to June was reduced due to reduced hospital activity and general national lockdown measures. As normal hospital activity resumed in July, the risk of transmission increased, therefore achieving zero in-hospital transmission in that period was a key target and achievement for the organisation.

Our staff feedback indicated that our choice of a combined Lean and person-centred approach meant that staff felt engaged and empowered. Feedback included:

- “It was good to hear what was happening outside my department–to know that others have the same worries as I do”;
- “lots of my team are worried about reopening–it is important that our opinions have been taken on board and listened to”;
- “lockdown has been isolating with teams not being able to meet. Collaborating on this project was good to bring wider teams together [even if this was on calls] and work together on a positive project.

A key unanticipated result was the wide cross organizational engagement in, and enthusiasm for, the improvement event. All departments participated in the voice of customer and process mapping exercises, resulting in more staff being exposed to Lean methodology. Following our work, interest in process improvement training opportunities increased across the hospital. Applications for funding to undertake the University Professional Certificate and Graduate Certificate in Process Improvement in Health Systems programmes increased fivefold. By recognizing the key role that qualified process improvement practitioners had played in the RIE, the study site increased funding for training opportunities and supported more staff in their completion of training in process improvement.

## 4. Discussion

The hospital successfully transitioned to “normal service” in July 2020. All departments reopened, offering full services. As detailed, this rapid improvement event was underpinned by the application of a person-centred approach, using CIP principles and actively seeking engagement [17,43]. There was widespread enthusiasm for returning to normal service. Stakeholder engagement sessions quickly revealed that there was also a very clear “perceived personal risk”. Team members were very concerned about the risk of increasing transmission rates between staff, patients and even their own families. Pushing through an improvement without listening, respecting and co-creating solutions to these concerns would not have been successful. One person or team did not have the answer to how to reopen the hospital safely. Hence, empowering all staff in cross-functional teams involved in a process to identify barriers and co-design solutions was the key to success. Our combined Lean and person-centred approach meant that staff rather than management led on the initiative (a bottom-up approach) which has been shown to leave staff feeling empowered [21,61,62]. Staff had a say in the nature of and direction of this project, which also made them feel empowered [63]. We recognized from the start that an important part of any improvement was staff satisfaction with their engagement in improvement initiatives [64,65].

By using the resumption of a normal activity plan to apply the same process across all hospital departments, each staff member had the opportunity to take an active role in planning the reopening and had the ability to discuss fears and suggest solutions. This removed the “them and us” or “tops and bottoms” roles [66]. No one department was expected to create solutions, nor was any department excluded. A sharing of knowledge across departments allowed for novel solutions to simple problems, for example, IT assisted in creating the electronic pre-appointment COVID-19 screen questionnaire, implemented by patient services. The success of this project has also helped solidify the improvement culture in the organisation. A shift has been noted, where previously, the process improvement practitioners would offer to assist in projects, following the service resumption project, their help has been actively requested. The organisation had to recognize the context of implementing change in the challenging environment as well as the system-wide impact of change [66]. It was recognised that ultimate success, as outlined earlier, would be realized if we delivered a system-wide standard workflow to resume normal activity. A key component of RIE is evaluating its effectiveness post-feedback. We undertook this evaluation using the “what works, what does not work framework” [41]. Feedback from all staff indicated that the digital COVID-19 screening questionnaire and access to PPE worked well. The process for urgent requests for pre-operative COVID-19 swabbing was more challenging, and perhaps this reflected the complexity of an emerging medical situation, specifically, empowering staff to facilitate urgent COVID-19 swabs and balancing the system-wide impact of these urgent requests—specifically for the laboratory.

The organisation response to resuming normal activity post-COVID-19 intentionally drew upon the three key theoretical areas of person-centredness [16], Lean Six Sigma [21] and co-design [43]. The project group that was convened was recognized as important within the local pandemic response and became a key group in the hospital’s response to COVID. This further evolved into a new approach that recognised the power of collaborative, inclusive and participatory ways of working and was more of a network than a project team. This allowed for a less formal approach but encouraged front line engagement. The network approach allowed an appropriate approach to a complex issue—that of achieving our goals whilst facilitating person-centred care for all. Following the end of the first wave of the pandemic, we decided to capitalize on this and develop new co-designed person-centred approaches to support the process of returning to normal activity. Our knowledge of the literature relating to person-centredness, Lean Six Sigma and co-leadership, informed and underpinned our strategic approach.

A key strength of this project was the existing UCD Academy and the availability of qualified, multidisciplinary process improvement practitioners within the study site. This allowed a top-down–bottom-up structure with support from all levels, from executive management to frontline staff to co-create solutions. Jones and Woodhead [67] similarly suggest that the “nurturing” of staff is best supported by other colleagues acting as mentors or coaches and not by delegating the implementation of improvement to external or internal consultants, which staff viewed negatively [68]. Staffs’ willingness to engage in the RIE was particularly impressive given during COVID-19, healthcare staff internationally had become exhausted, working long shifts, while facing extra sources of stress and anxiety [69].

We recognize that there are limitations to this project. Staff were operating in an unprecedented pandemic and were faced with a wide range of ethical dilemmas, where there was the extraction of people important in their patients’ lives and a potential to need to ration services, care and medical equipment. This may have influenced staff willingness to engage with the project, which promised a “return to normal”. However, Rychen & Salganik [70] claim that competence in leading and managing is best understood as the ability to work with others to meet challenges that arise in complex human systems—and staff certainly rose to the challenge. Another limitation we note is that the transferability of our findings is influenced by how kaizen practices were adopted at the studied hospital and the contextual factors therein. However, we assert that the use of the Lean to guide the resumption of normal activity has learning for other clinical sites. Another limitation we found was that the RIE did not allow time for a more detailed qualitative voice of the customer, which the hospital might have undertaken as part of a wider LSS project. In hindsight, a DMAIC framework, although not rapid, would have facilitated more detailed customer voice mapping, which has routinely been undertaken at the study site as part of its continuous improvement [32,33,34,35,71] However, due to the special cause circumstance of the COVID-19 pandemic [1,2,3] as described, we utilised a Lean RIE approach.

We recognize that, as this was a study within an unprecedented pandemic and in a single study site, we could only examine the feasibility of the RIE approach taken in the study and within the study site. The results do not necessarily generalize beyond the criteria of the study site. However, as pilot studies are conducted to evaluate the feasibility of some crucial component(s) of a full-scale study, we believe it has implications for other hospital sites and their academic partners who may wish to employ RIE using person-centred approaches in managing and responding to further COVID-19 surges.

## 5. Conclusions

Systems thinkers have an awareness of competence as a function of relationships, systems and culture [72,73,74]. Oshry (2007) suggests that senior executives can become overburdened by unmanageable complexities; frontline workers may feel vulnerable and neglected by authority figures whom they see as insensitive to the requirements of their jobs and middle managers feel pulled in opposing directions [29]. Our project highlighted how the study site used a combined Lean and person-centred approach to navigate this complexity in its response to COVID. Dixon Woods [75] suggests that the study of quality improvement methodologies in healthcare contributes to, and is important in developing an evidence-base, in looking at more than improvement interventions alone. This paper indicates that it is not just the outcomes of this project that were important but the involvement and participation of staff—the respecting of their worries and fears, as well as distributing the authority and power to construct solutions. For this project, we were fortunate to work with a team of process improvement practitioners who were also aware of the concepts and principles of person-centredness and who were open to collaborative working based around them. The co-design of solutions with staff was extremely important as the improvement required significant local understanding of, and reflection on, the pre-COVID-19 patient journeys to design the new patient journey. This echoes Oshry’s position that, to understand the entire system, it is necessary to adopt the position of a participant-observer who can stand apart from the whole system and observe it anew [74]. In addition to the application of RIE methodology in a healthcare setting, the importance of organisation-wide adoption of a person-centred approach to a critical situation is clear in this paper.

Successfully returning a private hospital to “normal” activity following the first COVID-19 lockdown was a challenging process. Introducing organisation-wide change is never easy and takes time (which was not available in this project setting). Complicating the project further was a “personal” factor—the fear of team members regarding COVID-19 transmission and spread among patients, colleagues and their family members. Lean and Six Sigma tools, including the voice of the customer, prioritizing solutions and mapping the desired future state provided clear frameworks for reopening departments. The utilisation of a person-centred approach ensured engagement and ownership among all staff in successfully delivering a resumption of routine activity. An additional unanticipated result of this rapid improvement event was the increased awareness and interest in process and quality improvement across the organisation. The hospital management and the hospital improvement team will continue to build on this enthusiasm to further imbed a culture of person-centred improvement.

## Figures and Tables

**Table 1:** Key Improvement tools used in RIE.

Tool	Definition	Purpose
**SIPOC** [18]	The high-level view of the process with SIPOC standing for Suppliers, Inputs, Processes, Outputs, Customers	Identify linkages between suppliers, customers, inputs, outputs and processes.
**Voice of the Customer** [39,40]	Understanding customer value and expectations	Understand expectations, success factors and key measures of staff, management, patients
**PICK chart** [35]	Used to classify and prioritise improvements reviewed–what is Possible, Implementable, a Challenge or Kill	Identified easy wins versus more complex solutions.
**Process Mapping** [35]	Process mapping (PM) supports a better understanding of complex systems and adaptation of improvement interventions to their local context.	Agree on the as-is process and opportunities for understanding bottlenecks and implementing improvements

**Table 2:** Service resumption rapid improvement event planner.

	Before (D1–D2)		During (D3–D6)				After (D7)
	D1	D2	D3	D4	D5	D6	D7
What? Define the problem	Resume Hospital operations in line with government guidance following COVID-19 Lockdown	Communicate project brief to stakeholders	Process mapping		Develop/prioritise solutions		Implementation plan agreed
How	Identify who should be involved in the team and stakeholders What are the measures of success? What restrictions in terms of project operations are to be considered secondary to COVID-19	– small group sessions, virtual, 1:1, email/survey	Virtual or physical project mapping facilitated by LSS practitioners involving nominated staff across all hospital departments.		Design session with IT to develop Pre attendance screening questionnaire. Trial of the patient journey through inpatient and outpatient areas		Priority tasks agreed and submitted to stakeholders and management.
Result	Target agreed Project team agreed Metrics agreed	Gather voice of customer theme areas of concern/opportunity. Create SIPOC	Desired state process maps validated by expert stakeholders and by department managers and staff		Spec for Electronic screening questionnaire complete Organisation-wide process for hospital attendance		Resumption plan agreed

**Table 3:** Membership and project role of resumption of service project team.

Position	Project Role	Expertise
Chief Operating Officer	Executive Sponsor	Overview of entire hospital operations
Allied Therapy Manager	Lean Practitioner/Process owner	Use of Lean methodologies
IPC manager	Expert stakeholder	IPC requirements
Head of Surgical Services	Expert stakeholder	Surgical patient pathway
Clinical Nurse manager inpatient acute ward	Expert stakeholder	Inpatient pathway and discharge process
Patient Services Manager	Expert stakeholder	Pre-appointment process
Head of Procurement	Expert stakeholder	PPE purchasing and distribution

**Table 4:** SIPOC of attendance for a hospital appointment.

Supplier	Input	Process	Output	Customer
Patient Clinical Staff Patient Services Team Consultants Scheduling Laboratory Infection Prevention Control Procurement Health and Safety Support Staff	Services and scheduling teams book and schedule patient appointments Clinical staff assess, diagnose and treat patients Laboratory run diagnostics Procurement ensure supply of materials (e.g., PPE) Health and Safety and Infection Prevention Control ensure standards are enacted and compliance with a safe environment of care Support Staff across all services–courtesy, security, catering, waste management, cleaning.	1. Appointment Booked 2. Text message reminder 3. Patient attends 4. Assessment/diagnostics/ Treatment/therapy/discharge 5. Patient invoiced 6. Follow up appointment booked 7. Clinical areas cleaned intra-use 8. Clinical areas ready for next patient 9. Patient discharged	Patient assessment, diagnostics, treatment/therapy/outcome Compliance with all Infection Prevention and Control guidelines Patient flow–capacity flexed to meet resumed normal service demand A skilled multidisciplinary team of staff available to deliver excellent care	Patients Staff The organisation Health Service Executive (HSE) Department of Health

**Table 5:** Results post-resumption of services in June 2020.

Metric	2019	2020	Meets Target
Inpatient occupancy	88%	75%	Yes–meets 75% target
Number of surgeries	17,378	15,377	Yes–exceeds 75% target
Number of outpatient visits	112,906	124,362	Yes -despite 3 months of reduced activity 2020 visits exceed 2019
Patient satisfaction	93%	95%	Yes
Inpatient COVID-19 transmission (July to December 2020)	Not applicable	0	Yes

## Data Availability

The data from this study is available in and presented as part of this paper.
